# β-HPV Infection Correlates with Early Stages of Carcinogenesis in Skin Tumors and Patient-Derived Xenografts from a Kidney Transplant Recipient Cohort

**DOI:** 10.3389/fmicb.2018.00117

**Published:** 2018-02-05

**Authors:** Cinzia Borgogna, Carlotta Olivero, Simone Lanfredini, Federica Calati, Marco De Andrea, Elisa Zavattaro, Paola Savoia, Elena Trisolini, Renzo Boldorini, Girish K. Patel, Marisa Gariglio

**Affiliations:** ^1^Virology Unit, Department of Translational Medicine, Novara Medical School, University of Eastern Piedmont, Novara, Italy; ^2^School of Biosciences, European Cancer Stem Cell Research Institute, Cardiff University, Cardiff, United Kingdom; ^3^Virology Unit, Department of Public Health and Pediatric Sciences, Turin Medical School, University of Turin, Turin, Italy; ^4^Dermatology Unit, Department of Health Sciences, Novara Medical School, Novara, Italy; ^5^Pathology Unit, Department of Health Sciences, Novara Medical School, Novara, Italy

**Keywords:** β-HPV, skin cancer, xenograft, carcinogenesis, organ transplant recipients

## Abstract

Many malignancies that occur in high excess in kidney transplant recipients (KTRs) are due to viruses that thrive in the setting of immunosuppression. Keratinocyte carcinoma (KC), the most frequently occurring cancer type in KTR, has been associated with skin infection by human papillomavirus (HPV) from the beta genus. In this report, we extend our previous investigation aimed at identifying the presence of active β-HPV infection in skin tumors from KTRs through detection of viral protein expression. Using a combination of antibodies raised against the E4 and L1 proteins of the β-genotypes, we were able to visualize infection in five tumors [one keratoacanthoma (KA), three actinic keratoses (AKs), and one seborrheic keratoses (SKs)] that were all removed from two patients who had been both transplanted twice, had developed multiple KCs, and presented with a long history of immunosuppression (>30 years). These infected tissues displayed intraepidermal hyperplasia and increased expression of the ΔNp63 protein, which extended into the upper epithelial layers. In addition, using a xenograft model system in nude mice displaying a humanized stromal bed in the site of grafting, we successfully engrafted three AKs, two of which were derived from the aforementioned KTRs and displayed β-HPV infection in the original tumor. Of note, one AK-derived xenograft, along with its ensuing lymph node metastasis, was diagnosed as squamous cell carcinoma (SCC). In the latter, both β-HPV infection and ΔNp63 expression were no longer detectable. Although the overall success rate of engrafting was very low, the results of this study show for the first time that β-HPV^+^ and ΔNp63^+^ intraepidermal hyperplasia can indeed progress to an aggressive SCC able to metastasize. Consistent with a series of reports attributing a causative role of β-HPV at early stages of skin carcinogenesis through ΔNp63 induction and increased keratinocytes stemness, here we provide *in vivo* evidence that these events are also occurring in the affected skin of KTRs. Due to these β-HPV-driven molecular pathways, the nascent tumor cell is able to acquire a high enough number of carcinogenic insults that its proliferation and survival will eventually become independent of viral gene expression.

## Introduction

The prolonged use of immunosuppressive drugs in kidney transplant recipients (KTRs) is a recognized risk factor for infectious diseases and ensuing complications, including infection-related malignancies (Vajdic and van Leeuwen, 2009; Krynitz et al., 2013; Geissler, 2015). Keratinocyte carcinoma (KC), which includes basal cell carcinoma (BCC) and squamous cell carcinoma (SCC), is the most frequent cancer type in KTRs, with an incidence rate ranging from 10 to 30% at 10 years post-transplantation (Kovach and Stasko, 2009; Madeleine et al., 2012).

Cutaneous human papillomaviruses (HPVs) from the β-genus, such as HPV5 and HPV8, have long been associated with KC in patients with a specific genetic background and in long-term immunosuppressed individuals (Genders et al., 2015; Howley and Pfister, 2015; Quint et al., 2015). Currently, more than 200 different HPV types have been described, which can be clustered into five genera (i.e., alpha-, beta-, gamma-, mu- and nu-papillomavirus) based on their taxonomy (http://pave.niaid.nih.gov/#home) (Bernard, 2013; Hufbauer and Akgül, 2017; Tommasino, 2017). Among them, mucosal α-genus HPV types are able to infect both oral and genital mucosa epithelia and are generally ranked as low-risk and high-risk types according to the tendency of the resulting neoplasm to undergo malignant progression (Kranjec and Doorbar, 2016). On the other hand, cutaneous HPVs, which are clustered in the evolutionarily distinct β-genus, can cause widespread unapparent or asymptomatic infections in the general population (Howley and Pfister, 2015; Quint et al., 2015). However, in immunosuppressed patients and in individuals suffering from the rare inherited disease epidermodysplasia verruciformis (EV), these viruses can spread unchecked and promote the development of KC. For example, β-HPV-associated wart-like lesions in EV patients are at high risk for progression to KC (Borgogna et al., 2012; Landini et al., 2014), and the ensuing tumors are positive for β-HPV DNA and protein expression (Dell'Oste et al., 2009).

Unlike cancers associated with mucosal HPV infections, cutaneous HPV-associated KC arises mainly in sun-exposed body areas, and hence these viruses are thought to cooperate with UV to induce cancer (Bouwes Bavinck et al., 2010, 2017; Quint et al., 2015). A series of reports have indeed demonstrated that expression of either β-HPV5 or 8 E6 substantially increases the mutagenic potential of UVB exposure (Wallace et al., 2012). For example, HPV8 E2, E6, and E7 each have oncogenic activities when expressed in basal epithelial cells of transgenic mice (Schaper et al., 2005; Pfefferle et al., 2008; Marcuzzi et al., 2009; Heuser et al., 2016). Furthermore, β-HPV E6s can alter Notch-mediated keratinocyte differentiation, thereby fostering a cellular environment conducive to viral replication (Meyers et al., 2013; Wallace and Galloway, 2014).

Although recent publications have, for the most part, characterized *in vitro* the effects of β-HPV oncogenes on host signaling pathways, it has become increasingly apparent that the dissection of the mechanisms underlying viral pathogenesis and oncogenesis in the affected skin areas during long-term HPV persistence would further our understanding of how such tissues are primed for cancer progression. Unfortunately, the exquisite host specificity of HPVs, which prevents experimental infections of heterologous hosts, has so far hindered the development of tractable animal models of infection. Moreover, it appears that β-HPV sequences are not essential for the maintenance of the transformed state as they are apparently lost from cancer cells, especially in non-EV tumors, at later stages of disease (Weissenborn et al., 2005).

In an effort to correlate β-HPV infection with disease pathology during progression of premalignant lesions to KC, we have set up an immunofluorescence protocol that allows both visualization of productive HPV infection in affected skin areas and evaluation of skin homeostasis through detection of the minichromosome maintenance complex component 7 (MCM7), a cellular proliferation marker (Borgogna et al., 2012, 2014). Using this protocol, in a previous study of a single center KTR cohort (Borgogna et al., 2014), we could observe active β-HPV infection in hyperplastic edges of skin SCC and BCC as well as in premalignant lesions, such as actinic keratoses (AK). Of note, while the E4^+^ areas were mainly found within the disorganized epithelium of AK lesions, in more advanced tumors, such as skin SCC and BCC, they were always localized in the adjacent pathological epithelium. Furthermore, we detected MCM7 expression extending into the upper epithelial layers of E4 expressing areas (E4^+^), indicating that epithelial cells were actively proliferating in areas of productive viral infection. Thus, based on these findings, we hypothesized that β-HPV-induced basal cell proliferation in the immunosuppressed setting alongside other transforming agents, such as UVB irradiation, might play a causative role at early stages of neoplastic transformation, while it may not be necessarily involved at later stages of the disease (Quint et al., 2015).

Emerging evidence also indicates that β-HPV can perturb p63 expression, a master regulator of development and maintenance of multilayered epithelia, including the epidermis (Meyers et al., 2013; Marthaler et al., 2017). p63, predominantly the ΔNp63 isoform, is selectively distributed in basal cells of stratified epithelia where it is required for the replenishment of stem cells (Senoo et al., 2007; Romano et al., 2009; Koster, 2010; Melino et al., 2015). In this regard, we have recently shown that HPV8-induced field cancerization in a transgenic mouse model is due to Lrig1^+^ keratinocyte stem cell expansion, which is accompanied by aberrant nuclear p63 expression in the expanded infundibulum and adjoining interfollicular epidermis. EV and non-EV keratoses displayed similar histology associated with β-HPV reactivation and nuclear p63 expression (Lanfredini et al., 2017).

In this study, we have further extended our previous analysis of a single center cohort of KTRs by (i) analyzing 128 additional skin lesions excised from 29 KTRs; (ii) correlating ΔNp63 expression levels with those of E4 and MCM7 in skin areas of productive HPV infection; and (iii) establishing and characterizing patient-derived xenografts as models to study skin cancer progression of β-HPV-infected skin lesions. In particular, we provide evidence of two KTRs with multiple β-HPV^+^ skin tumors and demonstrate progression of a β-HPV^+^ AK lesion into SCC in a patient-derived xenograft.

## Materials and methods

### Sample collection

Tissue sections were obtained from 171 formalin-fixed and paraffin-embedded blocks (FFPE) corresponding to either the core or the edges of the tumor, collected from 128 skin lesions excised from 29 KTRs attending the dermatology unit in the University Hospital-Novara. Thirty-two tumors were derived from KTRs already described in our previous work (patients 1M, 3M, 9M, 14M, 15M, and 17M respectively; Borgogna et al., 2014).

The study was approved by the local ethic committee: Comitato Etico Interaziendale Novara (Prot. CE 168/15) and written informed consent was obtained by all subjects according to the Declaration of Helsinki.

### Transplantation of human skin tumors

The immunodeficient mice used for xenograft development were male athymic Nude-Foxn1nu (Envigo, Huntingdon, UK). Mice were housed under pathogen-free conditions in our animal facilities in accordance with The Guide for the Care and Use of Laboratory Animals and the experimentation was approved by the CESAPO (Comitato Etico Sperimentazione Animale Piemonte Orientale, Prot. 20093). Mice were anesthetized with an Ohmeda TEC4 Vaporizer using Isoflurane Vet (Merial, Ingelheim am Rhein, Germany) and Gelfoam dressing (Johnson & Johnson, New Brunswick, NJ) was implanted into the dorsal sub-cutaneous space together with primary human fibroblasts suspended in 100 μl Matrigel (BD Biosciences, Milano, Italia), and wounds were closed with surgical stitches (Ethicon, Johnson & Johnson, New Brunswick, NJ). After 14 days, mice were anesthetized and human skin tumors (~0.5 cm^3^) were xenografted into the stromal bed together with 10^6^ primary human fibroblasts suspended in 100 μl Matrigel (Patel et al., 2012). Xenograft growth was weekly monitored by measuring the longer diameter of the tumor mass by caliper. After 3 or 6 months, mice were sacrified *via* CO_2_ inhalation and tumors were removed: half of them was fixed in 10% neutral-buffered formalin and embedded in paraffin blocks, while the other half was snap frozen and stored at −80°C. Five-μm sections were cut from the FFPE blocks and stained with hematoxylin and eosin (H&E) as performed for human skin biopsies.

### HPV genotyping

Total DNA was extracted from the frozen xenograft or plucked hair bulbs from eyebrows using QIAamp DNA Mini Kit following the manufacturer's instructions (Qiagen, Milan, Italy). β-HPV DNA analysis was carried out using broad spectrum PCR (PM-PCR) in combination with a reverse hybridization system [RHA Kit Skin (beta) HPV (Diassay, Rijswijk, the Netherlands)] as previously described (de Koning et al., 2006).

### Immunofluorescence labeling

DNA-FISH and immunofluorescence detection of β-HPV E4, MCM7, β-HPV L1 were performed on formalin-fixed and paraffin-embedded skin biopsies or xenografts using standard techniques as previously described (Borgogna et al., 2012, 2014). Briefly, DNA-FISH was performed using as probe the complete biotin-labeled (Biotin-nick translation kit, Roche Diagnostics GmbH, Mannheim, Germany) genomic HPV plasmid DNA (100 ng/ml). For the other markers analyzed (β-HPV E4, MCM7, β-HPV L1, and ΔNp63), antigen unmasking was performed by heating the slides in a conventional decloaking chamber for 10 min at 121°C in 10 mM citrate buffer at pH 6.0 (Vector Laboratories, Burlingame, CA, USA). Immunofluorescence was carried out using the following antibodies: rabbit anti-β-HPV E4 (kindly provided by John Doorbar, 1:1,000), mouse anti-MCM7 (1:200, Neomarkers Fremont, Fremont, CA), rabbit anti-ΔNp63 antibody (clone Poly6190) (BioLegend, San Diego, CA, USA) (1:500) and rabbit anti-β-HPV L1 (1:1,000). Images were acquired using a digital scanner (Pannoramic MIDI; 3D Histech Kft., Budapest, Hungary). For the assessment of histological features, the slides analyzed by immunofluorescence were disassembled and stained with H&E. The production of polyclonal antibodies to β-HPV E4 (HPV5E4) and β-HPV L1 has been previously described (Borgogna et al., 2012, 2014). Briefly, the broad spectrum anti-L1 antibodies were raised against the highly conserved region between amino acid 200 and 300 of the L1 protein, while those against the HPV5E4 crossreact with many genotypes from species 1 only, including HPV8, 14, 20, 24, 25, and 36.

## Results

### Visualization of β-HPV viral protein expression in skin tumors from the KTR cohort

In our previous work, we had already analyzed 111 FFPE blocks obtained from 79 skin lesions of 17 patients from a cohort of KTRs for the presence of active β-HPV infection by immunostaining with anti-E4 and anti-L1 antibodies. Among those specimens, we could find E4^+^ areas in six FFPE blocks from four patients corresponding to four AK lesions and the adjacent pathological area of one SCC and one BCC (Table 1). In this study, we have analyzed 171 additional FFPE blocks from 128 skin lesions excised from 29 KTRs following the same procedure. Within this second group, two patients, who were not included in the previous survey, presented with multiple skin lesions some of which displayed areas of β-HPV infection.

**Table 1:** Overview of the skin biopsy blocks from the KTR cohort study examined by immunohistochemistry for the presence of β-HPV infection and grouped according to pathology diagnosis.

	***Seborrheic keratoses, n = 18***	***Keratoacanthoma, n = 16***	***Bowen's disease, n = 3***	***Actinic keratoses, n = 54***	***Basal cell carcinoma, n = 77***	***Squamous cell carcinoma, n = 39***	***Total Lesions, n = 207***
	***FFPE blocks n** = **20***	***FFPE blocks n** = **21***	***FFPE blocks n** = **3***	***FFPE blocks n** = **71***	***FFPE blocks n** = **107***	***FFPE blocks n** = **60***	***Total FFPE blocks n** = **282***
β-HPV-positive lesions	1	1		7	1	1	11
Previous study (Borgogna et al., 2014)				4	1	1	6
This study	1	1		3			5

*The skin biopsies found positive for β-HPV E4 expression in our previous study (Borgogna et al., 2014) and this current one are indicated separately*.

**Figure 1:**
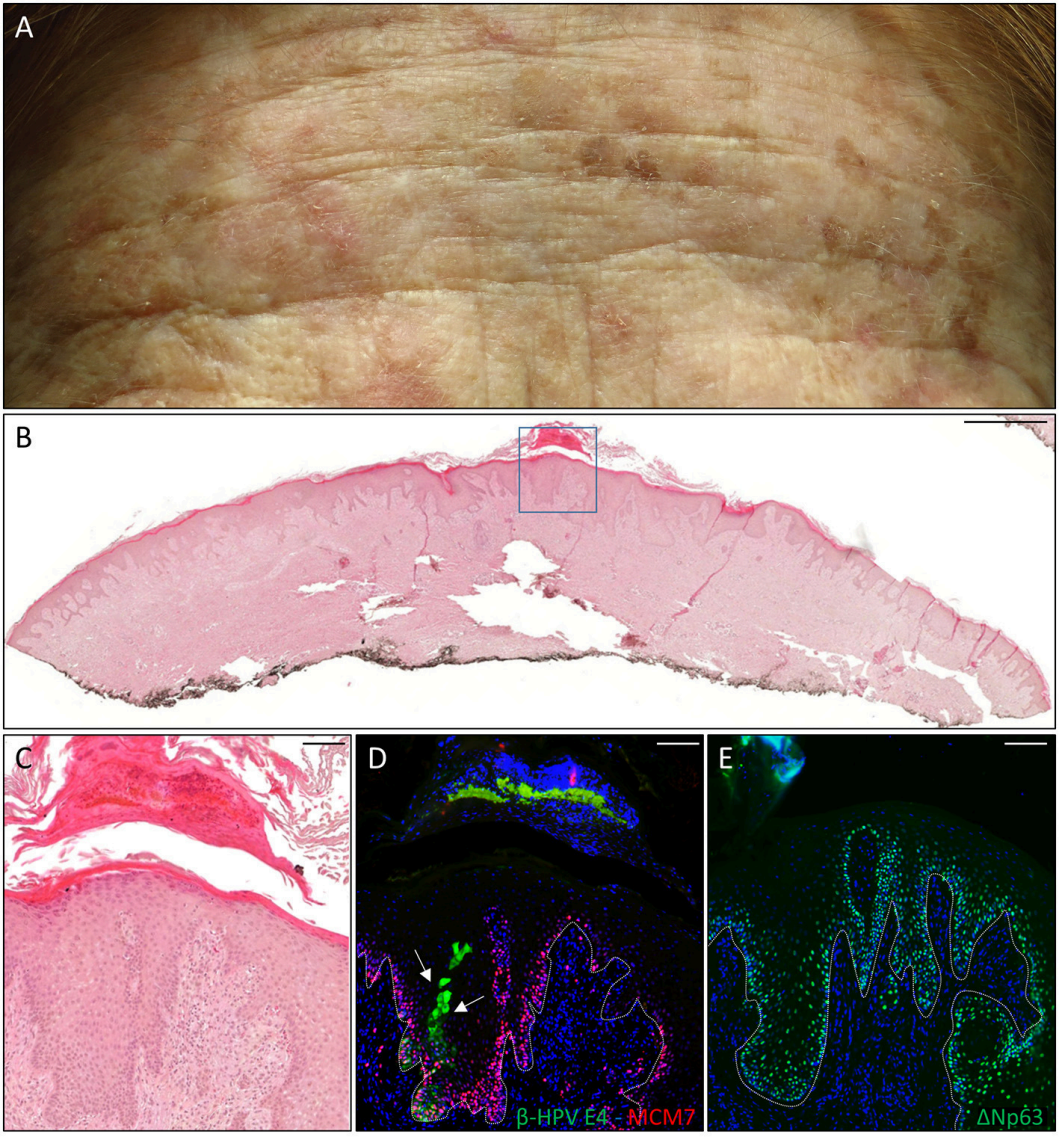
Histopathological analysis of an actinic keratoses (AK) from a female kidney transplant recipient (KTR). **(A)** Flat whitish and reddish papular lesions on her forehead. **(B)** Hematoxylin and eosin (H&E) staining of a representative tissue section (scale bar: 1,000 μm) from one AK lesion on the leg of the patient. **(C)** Magnification of the region highlighted by the blue box shown in panel B (scale bar: 100 μm). **(D)** Immunofluorescence staining for beta human papillomavirus (β-HPV) E4 and minichromosome maintenance complex component 7 (MCM7) (β-HPV E4 in green; MCM7 in red) of the same tissue section shown in **(B,C)**. The white arrows indicate E4 staining. **(E)** Immunofluorescence staining for ΔNp63 (green) of a serial section. The white dotted line indicates the basal layer. Sections in **(D,E)** were counterstained with 4′,6-diamidino-2-phenylindole (DAPI) (blue) to visualize cell nuclei.

The first case was a female KTR who underwent a second kidney transplant (KTx) in 2016 due to the failure of her first one after 25 years. She displayed many flat, reddish papular lesions widespread in the total body skin as shown by the representative picture of the forehead (Figure 1A) that were very much resembled the EV keratoses, and her clinical history reported the development of many proliferative skin lesions (>15) in different body sites starting approximately 10 years after her first kidney transplant, which was carried out in 1980. A total of 22 FFPE blocks corresponding to the 13 tumors excised from this patient from 2014 in our hospital, each diagnosed as SCC (*n* = 5), BCC (*n* = 3), AK (*n* = 2), or keratoacanthoma (KA) (*n* = 3), were made available for immunofluorescence analysis. β-HPV^+^ areas were found in one KA located on the leg, and in two AKs located on the hand and the leg (Table 1). As shown in representative (Figures 1C,D), E4^+^ cells were clearly evident in the areas of an AK lesion presenting with hyperkeratosis and parakeratosis; some of them were trapped in the keratin layers where L1^+^ nuclei were also observed (data not shown). The cellular proliferation marker MCM7, a subunit of DNA helicase, was more evident in the E4^+^ area compared with the adjacent epithelium, and extended to suprabasal layers, indicating that cells were primed to enter the cell cycle. In the AK found on the hand, E4 expression was not detected, while nuclei stained with the β-HPV late capsidic protein (L1) were well evident in the upper layers of the epithelium where some differentiation was still occurring, indicating the presence of productive viral infection (data not shown).

**Figure 2:**
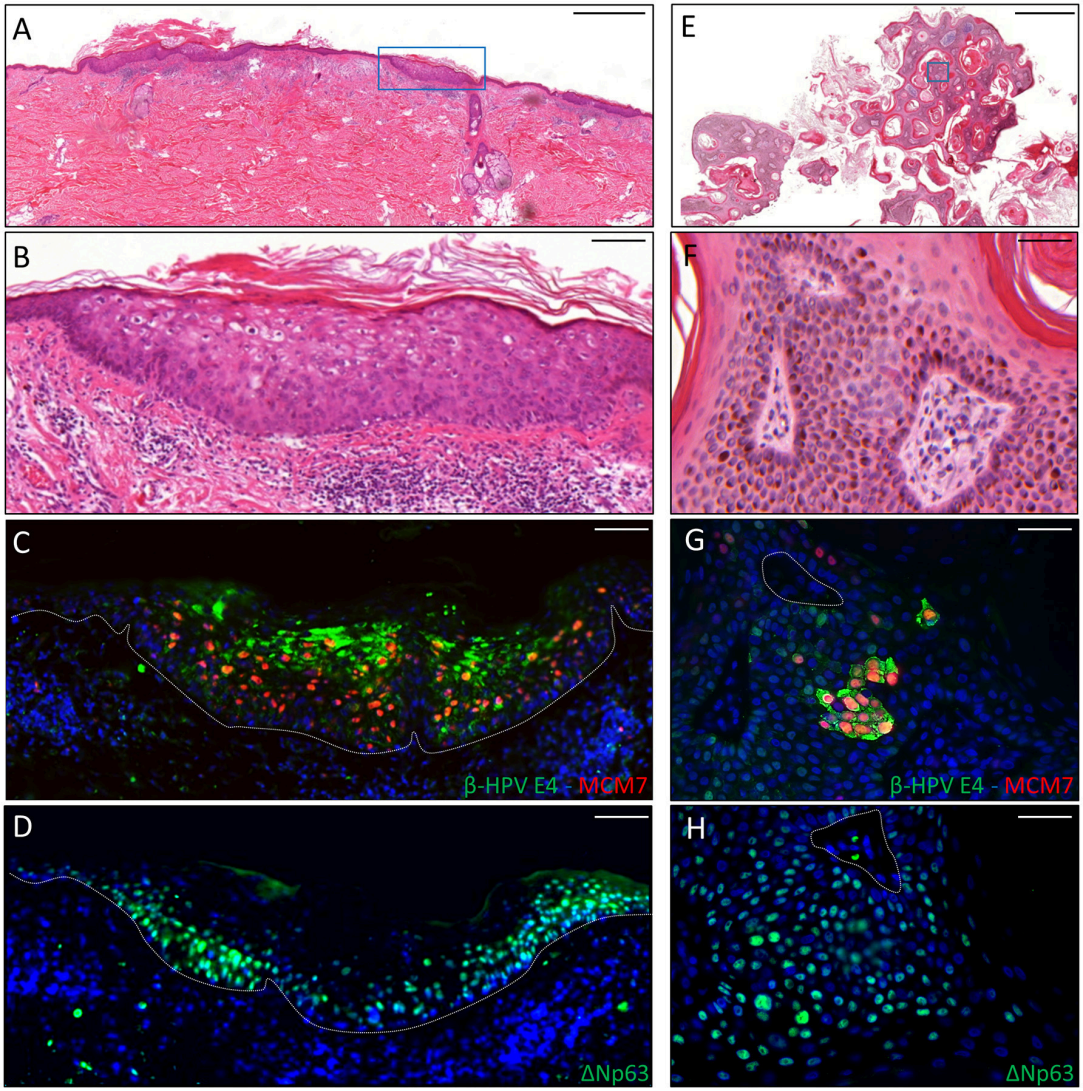
Immunohistochemical staining for β-HPV E4, MCM7, and ΔNp63 in one AK lesion **(A–D)** and one seborrheic keratoses (SK) lesions **(E–H)** derived from a male KTR. **(A)** H&E staining of a representative tissue section (scale bar: 1,000 μm) from one AK lesion on the back of the patient. **(B)** Magnification of the region highlighted by the blue box shown in **A** (scale bar: 100 μm). **(C)** Immunofluorescence staining for β-HPV E4 (green) and MCM7 (red) of the same tissue section shown in **(A,B)**. **(D)** Immunofluorescence staining for ΔNp63 (green) of a serial section. The white dotted line indicates the basal layer. **(E)** H&E staining of a representative tissue section (scale bar: 1,000 μm) from one SK lesion on the forearm of the patient. **(F)** Magnification of the region highlighted by the blue box shown in **E** (scale bar: 50 μm). **(G)** Immunofluorescence staining for β-HPV E4 (green) and MCM7 (red) of the same tissue section shown in **(E,F)**. **(H)** Immunofluorescence staining for ΔNp63 (green) of a serial section. Sections **(C,D,G,H)** were counterstained with DAPI (blue) to visualize cell nuclei.

The second case was a male KTR born in 1960 who underwent kidney transplant twice, with the first one taking place in 1996. From 2012 onwards, the patient developed five skin cancers in different body sites diagnosed as BCC (*n* = 2), AK (*n* = 2) and seborrheic keratoses (SK; *n* = 1). Positivity for β-HPV markers was found in one AK and in the SK removed from the back and the left forearm, respectively. The virus-positive AK displayed highly keratotic epithelium with clear signs of β-HPV-related cytopathic effects (Figures 2A,B). Immunofluorescence analysis showed large areas of β-HPV E4 expression in the most superficial layers of the epithelium and increased MCM7 expression in the basal and suprabasal layers (Figure 2C). Likewise, in the SK we found a large number of E4^+^ cells spread throughout the entire lesion as shown in Figures 2E–G. Consistent with the benign nature of this skin tumor, MCM7 expression in the basal layer was less evident, while it was highly expressed in the suprabasal cells overexpressing cytoplasmic E4. These cells located in the mid to upper epithelial layers had likely re-entered the cell cycle to support viral genome amplification as E4 expression usually occurs in cells where viral DNA is replicating.

### ΔNp63 expression in skin tumors

**Figure 3:**
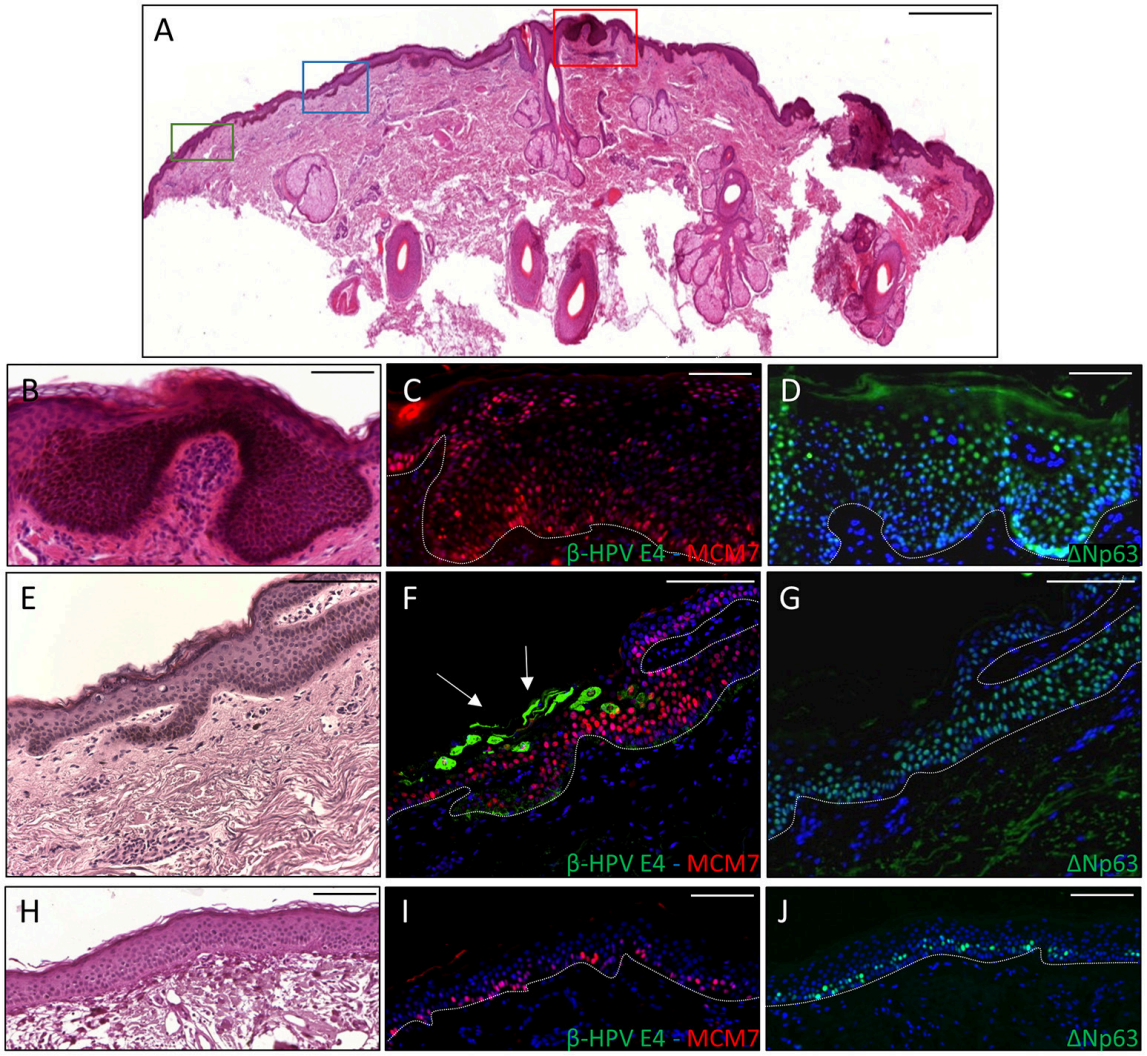
Immunohistochemical staining for β-HPV E4, MCM7, and ΔNp63 in one basal cell carcinoma (BCC) from a KTR (patient 5M, Borgogna et al., 2014). **(A)** H&E staining of a representative tissue section (scale bar: 1,000 μm). The red square highlights the BCC, the blue square indicates the HPV-infected area in the adjacent epithelium, and the green square shows non-pathological epithelium. **(B,E,H)** Magnification of the regions highlighted by the boxes shown in **A** (**B**: red square; **E**: blue square; **H**: green square). Scale bar: 100 μm. **(C,F,I)** Serial sections obtained from the same specimens depicted in panels B, E, and H, respectively, were subject to double staining using antibodies against β-HPV E4 (green) and MCM7 (red). The white arrows indicate E4 staining. **(D,G,J)** Serial sections obtained from the same specimens depicted in **(B,E,H)**, respectively, were stained with antibodies against ΔNp63 (green). The white dotted line indicates the basal layer. All sections were counterstained with DAPI (blue) to visualize cell nuclei.

To ascertain whether p63 induction could also be visualized in virus-positive skin cancers from the KTR cohort, tissue sections from all the skin lesions excised from the two aforementioned patients (*n* = 18) were co-labeled with antibodies against ΔNp63 and MCM7. In addition, six skin lesions (SCC *n* = 1, BCC *n* = 1 and AK *n* = 4) with areas positive for E4 expression from our previous survey were similarly analyzed. As depicted in the representative Figure 3 showing a KTR-derived BCC identified in the first round of screening (patient 5M, Borgogna et al., 2014), the ΔNp63 protein was highly expressed in the neoplastic area in comparison with the non-pathological adjoining epithelium (Figure 3D vs. Figure 3J), and its expression generally overlapped with that of MCM7 (Figures 3C,I respectively). This staining pattern was reproducibly observed in the high-grade lesions, including SCC (*n* = 6) and BCC (*n* = 6). Of note, in the adjacent β-HPV-infected hyperplastic epithelium as determined by E4 staining (Figure 3F), ΔNp63 expression extended in the middle-superficial layers, higher than those expressing MCM7, indicating that ΔNp63 was expressed in cells either proliferating or with a great potential of proliferation although not yet transformed. A similar pattern of ΔNp63 expression was also observed in the benign SK shown in Figure 2, where it spread across the entire epithelium despite the fact that MCM7 was restricted to some cells in the basal layer and some cells in the intermediate layers (Figures 2G,H). Also in the case of AK (*n* = 8) and KA (*n* = 3), ΔNp63 expression exceeded that of MCM7 as it was still detectable in the more superficial nucleated cells. Quantification of ΔNp63 and MCM7 positive cells confirmed the increase in ΔNp63^+^ cells in both β-HPV-infected cells and tumors vs. non-pathological epithelium (Supplementary Figure 1). In the case of β-HPV-infected cells, the number of ΔNp63^+^ cells was significantly higher than that of MCM7^+^ cells.

### Development of xenografts in athymic nude mice

A well-established model to study tumor progression and clinical outcome is represented by patient-derived xenograft (PDX) models. PDXs are developed by implanting tumor samples obtained directly from patients in immunodeficient mice. In this regard, we have previously shown that the creation of a humanized stromal bed, by first implanting Gelfoam dressing along with HDF, facilitates primary human skin SCC xenograft growth in nude mice (Patel et al., 2012).

**Figure 4:**
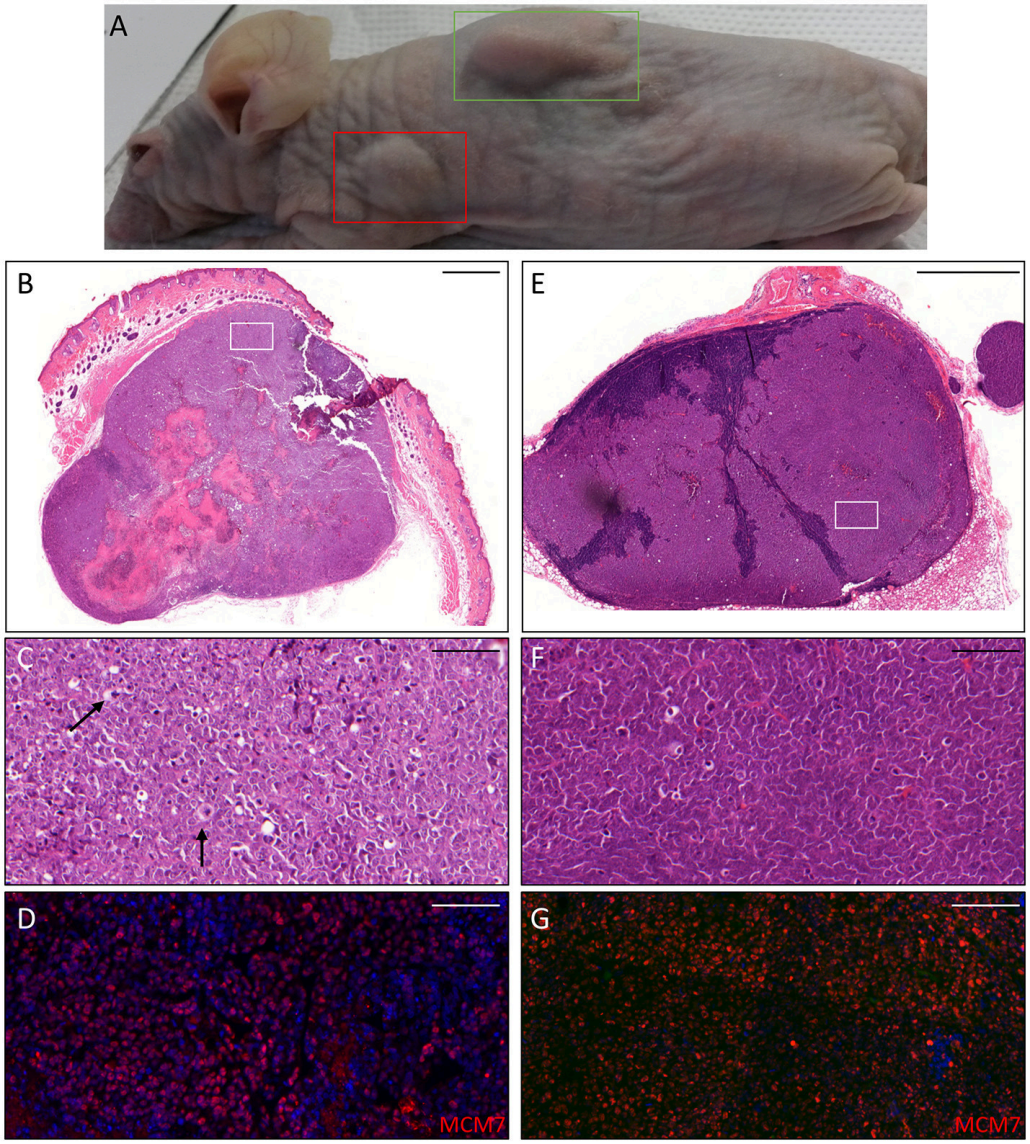
Histopathological analysis of an AK xenograft derived from the male KTR. **(A)** Nude mouse in which the xenograft was diagnosed as SCC (green box) giving rise to a lymph node metastasis (red box). **(B,E)** Histopathological features (H&E staining) of the lesions corresponding to the boxes highlighted in **A** (**B**: SCC; **E**: lymph node metastasis). Scale bars: 1,000 μm. **(C,F)** Magnification of the areas highlighted by the white boxes depicted in **(B,E)**. Scale bar 100 μm. The black arrows indicate cells in mitotic phase. **(D,G)** Immunofluorescence staining for MCM7 (red) of the same tissue section shown in the **(C,F)** respectively. Sections **(D,G)** were counterstained with DAPI (blue) to visualize cell nuclei.

**Figure 5:**
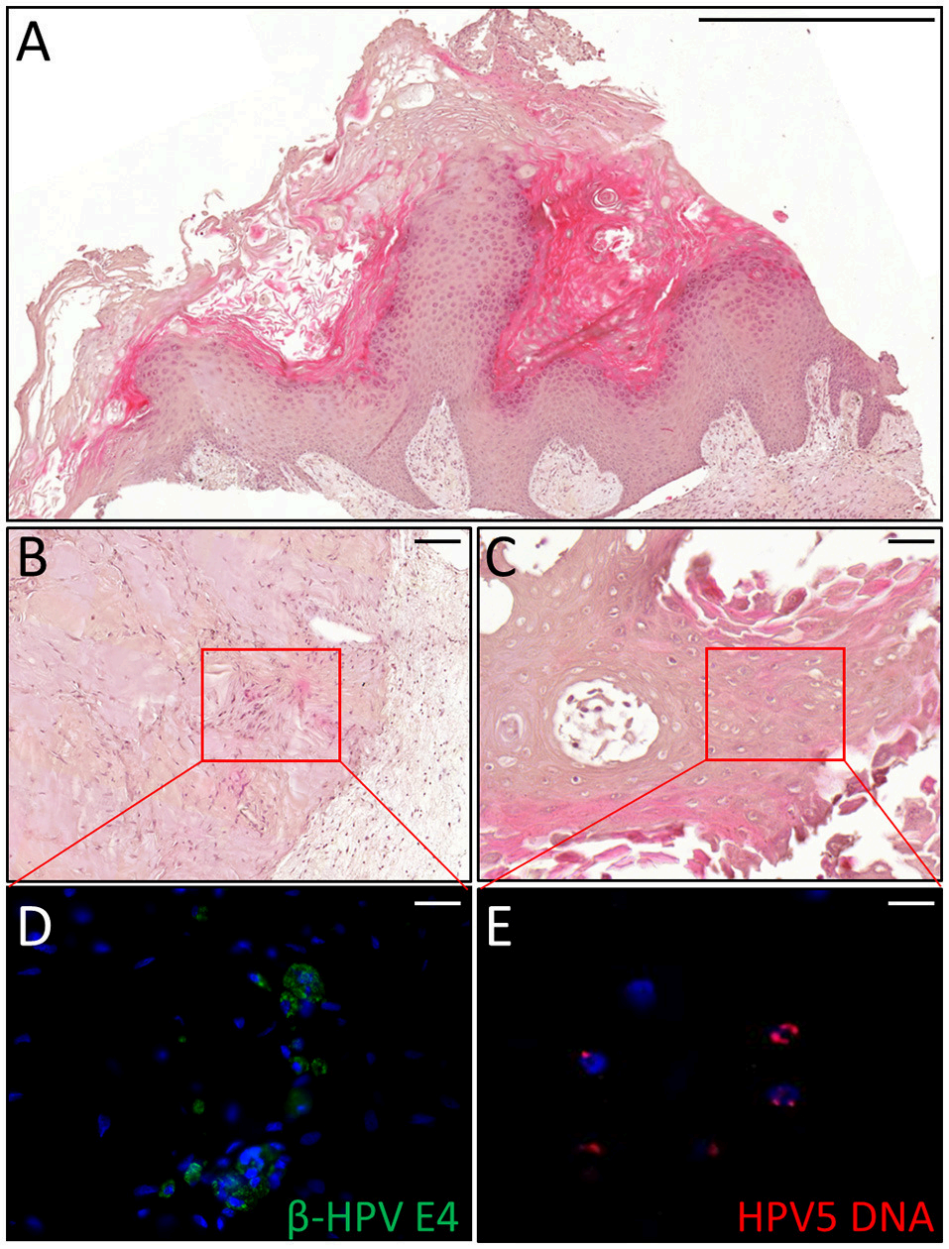
Histopathological analysis of an AK xenograft derived from the female KTR. **(A)** H&E staining of a representative sections of the xenograft. Scale bar 1,000 μm. **(B,C)** H&E staining of two representative areas positive for the viral markers. Scale bars: 100 μm. **(D,E)** Magnification of the areas corresponding to the red squares highlighted in the top pictures and immunostained for β-HPV E4 protein (green, **D**) and HPV5 DNA (red, **E**). Sections **(D,E)** were counterstained with DAPI (blue) to visualize cell nuclei. Scale bars: 10 μm.

Thus, we asked whether premalignant skin lesions could be similarly engrafted so that we could monitor progression or regression of a lesion that otherwise would be surgically removed from the patient. For this purpose, we engrafted all skin lesions available from the cohort study in the period of observation (3 years), including those that clinically appeared as benign and/or premalignant. From a total of 42 lesions engrafted following this procedure, we were able to collect 38 xenografts—in four cases no tumor growth was detected. The main characteristics of the KTRs whose skin tumors were grafted into nude mice are shown in Supplementary Table 1, and the histological features of the tumors are listed in Table 2. At the beginning, tumors were removed 6 months after the graft (15 out of 38). Following this timeline, growth was obtained for two BCCs whose xenograft histology closely mirrored that found in the original tumor. The human origin of the xenograft tumors was confirmed by staining with human cytokeratin (data not shown). Unfortunately, all other cases (*n* = 13) turned out to be epidermal cysts filled with cornified material and surrounded by dying epithelial cells. This pitfall prompted us to shorten the length of the growing period in nude mice to 3 months. Using this modified timeline, three out of the 23 skin tumors engrafted gave rise to a tumor mass with well-defined histological features. Three AKs successfully grew in nude mice; two of them displayed a growth curve similar to that of the two BCCs, while one grew faster, especially after week 8 (Supplementary Figure 2). While the formers xenografts retained the original histological features, the latter, which was derived from the aforementioned male KTR, was diagnosed as SCC along with its ensuing lymph node metastasis (Figure 4). Although β-HPV markers were well evident in multiple areas of the original AK (Figures 2A–D), they were no longer detectable in the SCC, not even by PCR amplification of xenograft total DNA using broad spectrum primers for beta genotypes (data not shown, de Koning et al., 2006). This SCC turned out to be negative for ΔNp63 expression by immunohistochemistry (data not shown), while it was strongly positive for MCM7. From the same patient, we also implanted the β-HPV^+^ SK shown in Figures 2E–H, which failed to grow in nude mice probably due to its benign nature. One of the two other AKs was from a patient transplanted in 1995 who had developed three AKs and one SCC after 20 years of transplantation. In this case, we failed to detect any viral protein expression in both xenograft and original tumor as it was the case in all the other skin lesions excised from this KTR (data not shown). The second AK was from the KTR female described above (Figure 1). Consistent with the fact that a documented β-HPV reactivation was reported in the female patient as well as in her original skin lesion, in this latter xenograft, we were able to detect in some cells both viral protein E4 expression and the presence of the HPV5 genome by FISH (Figure 5). The HPV5 probe was used because this genotype was detected in plucked hair bulbs from the eyebrows (data not shown), which are regarded as representative samples of the virus carriage on a person's skin. Furthermore, one SCC and one BCC were engrafted from the same patient, but they failed to grow in nude mice.

**Table 2:** Histological features of the skin tumors grafted into nude mice, timeline of excision and outcome of xenograft establishment.

	**No. of tumors (tot: 42)**	**Xenograft excision after**		**Xenograft establishment**	
		**3 months**	**6 months**	**Failure**	**Growth**
Squamous cell carcinoma	6	6	0	6	
Basal cell carcinoma	16	9	7	14	2 (BCC)
Keratoacanthoma	1	0	1	1	
Actinic keratoses	14	6	8	11	- 2 (AK) - 1 (SCC+ metastasis)
Seborrheic keratoses	5	3	2	5	

## Discussion

In this study, we extend our previous investigation aimed at identifying the presence of active β-HPV infection in skin tumors from KTRs through the detection of viral protein expression, such as E4 and L1 (Borgogna et al., 2014). In this new round of screening, we found positive staining in five tumors that were all removed from two patients who had been both transplanted twice and presented with a long history of immunosuppression (>30 years). The female patient had developed many skin cancers (>15) accompanied by the development of a clinical picture highly resembling that of an EV patient. Consistent with her clinical manifestations revealing diffuse actinic damage on her forehead and dorsum of the hands, β-HPV viral protein expression was observed in three skin tumors, corresponding to two AKs and one KA. Likewise, among the five skin tumors removed from the other male patient, one AK and one SK showed extensive E4 expression.

According to our previous findings, E4 expression was mostly detected at early stages of disease, confirming that virus expansion was taking place in the skin under condition of prolonged immunosuppression, albeit no longer detectable in more advanced stages of disease (Borgogna et al., 2012, 2014; Landini et al., 2014). These infected tissues displayed intraepidermal hyperplasia as demonstrated by histology, enhanced expression of the MCM7 proliferation marker in the suprabasal layers, and ΔNp63 expression. Intriguingly, ΔNp63 protein expression levels were also high in both benign and premalignant lesions as well as in cells that did not express MCM7 and, therefore, were not regarded as actively proliferating.

Overall, given the important role of p63 in the proliferation and maintenance of epidermal stem cells, and a number of *in vitro* and *in vivo* data linking β-HPV infection to ΔNp63 upregulation (Lanfredini et al., 2017; Marthaler et al., 2017), our findings further strengthen the proposed model of β-HPV-induced skin carcinogenesis in an immunosuppressed setting through p63 activation and increased keratinocyte stemness. Consistently, using a novel bitransgenic mouse model where ΔNp63 is overexpressed moderately in the basal layer of stratified epithelia, Devos et al. have recently demonstrated that overexpression of ΔNp63 favors early steps of skin tumorigenesis (Devos et al., 2017). Development of benign lesions, such as papilloma, was indeed accelerated following ΔNp63 overexpression in a dose-dependent manner in a classical two-stage chemical carcinogenesis protocol where an increased number of papilloma was observed.

Emerging evidence clearly indicates that β-HPVs contribute to skin cancer development at early stages and through molecular mechanisms that are different from those reported for high-risk α-types, such as HPV16 and 18 (Howley and Pfister, 2015; Quint et al., 2015). Our group and others have reported that β-HPV can increase stemness properties of keratinocytes in both monolayer cultures of keratinocytes transduced with HPV8 E6 and E7 viral proteins and an HPV8 transgenic mouse model where the skin-specific transcription of the early region of HPV8 is driven by the K14 promoter (Hufbauer et al., 2013; Lanfredini et al., 2017). In all these experimental models, as well as in β-HPV-infected human skin, this increased stemness is always accompanied by ΔNp63 overexpression (Marthaler et al., 2017). Thus, together with previous reports pointing to a possible cooperation between E6 expression and UVB exposure in skin carcinogenesis, our findings provide *in vivo* evidence that β-HPV infection might be a co-factor of early stages of skin cancer progression. This hypothesis is fully compatible with the “hit-and-run” mechanisms of carcinogenesis, with cutaneous β-HPV playing an important role for tumor initiation and progression, but not required for tumor maintenance. Assuming that viral replication is more active at very early stages of carcinogenesis, or when lesions are not yet even clinically evident and rarely removed, it seems reasonable to propose that at later stages of disease, when lesions require surgical excision, β-HPV infection is no longer detectable or otherwise restricted to some residual areas at the edges of the lesion. These assumptions can explain the low percentage of positivity found in our study. However, the observed reactivation of β-HPV infection occurring in the skin of KTRs alongside the demonstration that these viruses can increase keratinocyte stemness and interfere with the DNA damage response argues in favor of a model whereby the cooperation between β-HPV and UVB, which triggers skin carcinogenesis, is no longer required to maintain the malignant state at later stages of neoplastic development (Hufbauer and Akgül, 2017; Lanfredini et al., 2017; Wendel and Wallace, 2017).

Another important aspect of the current study is the optimization and implementation of a unique xenograft model system in nude mice (Patel et al., 2012). Thanks to the creation of a humanized stromal bed in the site of grafting, here we show that it is possible to obtain xenograft growth also from small biopsy samples from relatively benign skin lesions such as AKs. However, the overall success rate of this procedure was very low as only three out of 14 engrafted AKs grew as PDXs. Furthermore, we failed to grow any SCCs while two out of the 16 engrafted BCCs gave rise to a tumor mass with the same histological features of the original tumor. This low rate of success may be due to the low proliferating index of the tumor, especially for the AK, or the size of the engrafted tumor, which in some cases was small and very likely did not include enough cells with high proliferating capacity. Despite these limitations, the results of this study are extremely exciting because we show for the first time that β-HPV^+^ and ΔNp63^+^ intraepidermal hyperplasia can indeed progress to an aggressive SCC, including metastasis. As this SCC developed in 12 weeks in a nude mouse, we cannot rule out that the engrafted AK already harbored small amount of SCC. Nevertheless, cancer cells were originated from the infected epithelium that clinically appeared as field cancerization. Furthermore, the observation that both ΔNp63 and β-HPV are no longer detectable in the SCC suggests that their roles might be more important at premalignant stages where they likely cooperate to create an environment conducive to skin cancer progression.

Overall, the xenografts obtained following our protocol may prove useful not only in following the fate of each lesion but also in increasing the availability of biological samples for molecular investigations, which is generally limited due to surgical removal of premalignant lesions. In this regard, every effort should be made by clinicians involved in the daily management of KTRs to identify patients with clinically evident virus reactivation in the skin, and as such at increased risk of developing cancer.

## Author contributions

MG, MD and GP conceived the project. CB and CO designed experiments. CB, CO, SL, FC, RB, and ET performed experiments and collected, analysed data. EZ and PS enrolled patients and collected clinical samples. MG drafted the manuscript with inputs from all authors. CB and MG handled funding, supervision, and coordination. All authors have made final approval for the final version to be submitted.

### Conflict of interest statement

The authors declare that the research was conducted in the absence of any commercial or financial relationships that could be construed as a potential conflict of interest.
