# Supplementary material for: β-HPV Infection Correlates with Early Stages of Carcinogenesis in Skin Tumors and Patient-Derived Xenografts from a Kidney Transplant Recipient Cohort
Source: Front Microbiol. 2018 Feb 5;9:117. doi: 10.3389/fmicb.2018.00117 (PMC5807414; doi:10.3389/fmicb.2018.00117)
Supplement: Supplementary file 1 [file Table1.PDF]

Supplementary Materials

β-HPV Infection Correlates with Early Stages of Carcinogenesis in Skin Tumors and Patient-Derived Xenografts from a Kidney Transplant Recipient Cohort

Cinzia Borgogna[1*], Carlotta Olivero[1,2*], Simone Lanfredini[2], Federica Calati[1], Marco De Andrea[1,3], Elisa Zavattaro[4], Paola Savoia[4], Elena Trisolini[5], Renzo Boldorini[5], Girish Patel[2], and Marisa Gariglio[1].

[1] Virology Unit, Department of Translational Medicine, Novara Medical School, Italy
[2] European Cancer Stem Cell Research Institute, School of Biosciences, Cardiff University, UK
[3] Virology Unit, Department of Public Health and Pediatric Sciences, Turin Medical School, Italy
[4] Dermatology Unit, Department of Health Sciences, Novara Medical School, Italy
[5] Pathology Unit, Department of Health Sciences, Novara Medical School, Italy

*These authors contributed equally to this work.

Corresponding Author: marisa.gariglio@med.uniupo.it

Table 1S. Main characteristics of the KTRs whose skin tumors were grafted into nude mice.

		Patients (n) (tot: 25)	Lesions (n) (tot:42)
SEX	**Male**	21	36
	**Female**	4	6
AGE at surgery	**>60**	23	36
	**≤ 60**	2	6
Years from transplantation to surgery	**≤ 10**	13	20
	**10-20**	7	13
	**21-30**	0	0
	**>30**	5	9
