# Supplementary material for: β-HPV Infection Correlates with Early Stages of Carcinogenesis in Skin Tumors and Patient-Derived Xenografts from a Kidney Transplant Recipient Cohort
Source: Front Microbiol. 2018 Feb 5;9:117. doi: 10.3389/fmicb.2018.00117 (PMC5807414; doi:10.3389/fmicb.2018.00117)
Supplement: Supplementary file 2 [file Image1.pdf]

Supplementary Materials

β-HPV Infection Correlates with Early Stages of Carcinogenesis in Skin Tumors and Patient-Derived Xenografts from a Kidney Transplant Recipient Cohort

Cinzia Borgogna[1*], Carlotta Olivero[1,2*], Simone Lanfredini[2], Federica Calati[1], Marco De Andrea[1,3], Elisa Zavattaro[4], Paola Savoia[4], Elena Trisolini[5], Renzo Boldorini[5], Girish Patel[2], and Marisa Gariglio[1].

[1] Virology Unit, Department of Translational Medicine, Novara Medical School, Italy

[2] European Cancer Stem Cell Research Institute, School of Biosciences, Cardiff University, UK

[3] Virology Unit, Department of Public Health and Pediatric Sciences, Turin Medical School, Italy

[4] Dermatology Unit, Department of Health Sciences, Novara Medical School, Italy

[5] Pathology Unit, Department of Health Sciences, Novara Medical School, Italy

*These authors contributed equally to this work.

Corresponding Author: marisa.gariglio@med.uniupo.it

Figure 1S. Percentage of ΔNp63+ or MCM7+ epithelial cells in skin lesions from the KTR cohort (n= 24, FFPE n=36). Positive cells were counted on three random fields (20X magnification) for each area: Non-pathological epithelium, HPV-infected area, Tumor. Error bars, SD. *, $p < 0.05$.

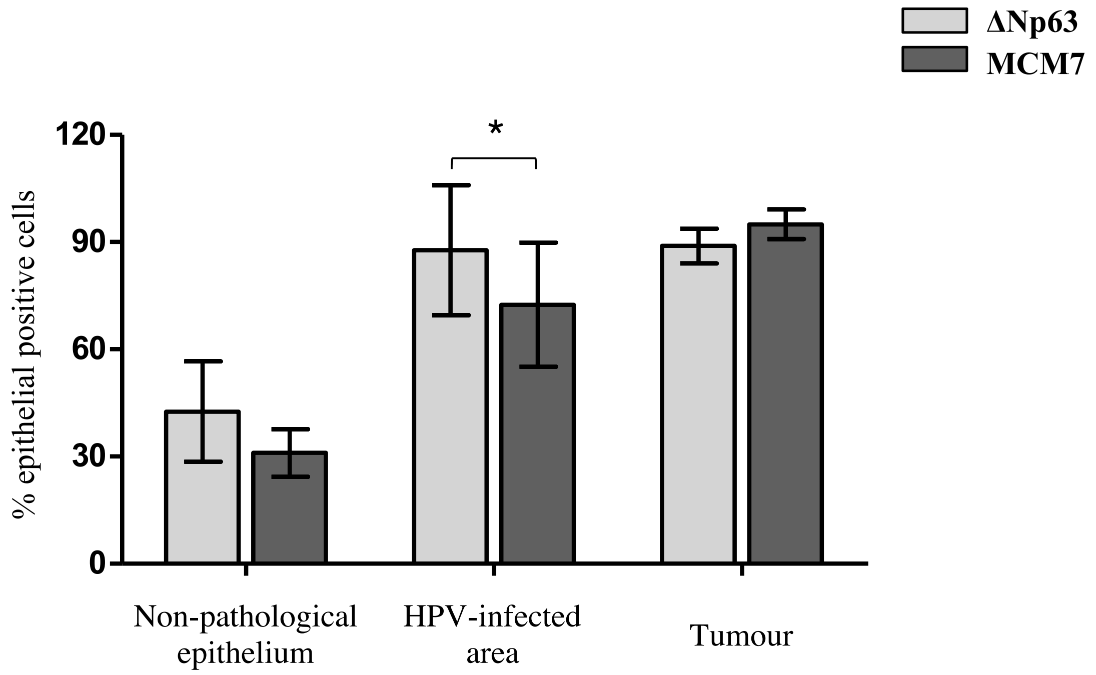

Supplementary Materials and Methods

Statistical Analysis

For ΔNp63+ and MCM7+ cell quantification, mean value ± one standard deviation was presented. The statistical significance between two experimental groups is indicated in the figure by an asterisk. Comparisons were made using the Student's t test. Calculated *p*-values less than or equal to 0.05 were considered to be of statistical significance.
