# Supplementary material for: β-HPV Infection Correlates with Early Stages of Carcinogenesis in Skin Tumors and Patient-Derived Xenografts from a Kidney Transplant Recipient Cohort
Source: Front Microbiol. 2018 Feb 5;9:117. doi: 10.3389/fmicb.2018.00117 (PMC5807414; doi:10.3389/fmicb.2018.00117)

Supplementary Materials

β-HPV Infection Correlates with Early Stages of Carcinogenesis in Skin Tumors and Patient-Derived Xenografts from a Kidney Transplant Recipient Cohort

Cinzia Borgogna[1*], Carlotta Olivero[1,2*], Simone Lanfredini[2], Federica Calati[1], Marco De Andrea[1,3], Elisa Zavattaro[4], Paola Savoia[4], Elena Trisolini[5], Renzo Boldorini[5], Girish Patel[2], and Marisa Gariglio[1].

[1] Virology Unit, Department of Translational Medicine, Novara Medical School, Italy

[2] European Cancer Stem Cell Research Institute, School of Biosciences, Cardiff University, UK

[3] Virology Unit, Department of Public Health and Pediatric Sciences, Turin Medical School, Italy

[4] Dermatology Unit, Department of Health Sciences, Novara Medical School, Italy

[5] Pathology Unit, Department of Health Sciences, Novara Medical School, Italy

*These authors contributed equally to this work.

Corresponding Author: marisa.gariglio@med.uniupo.it

Figure 2S. Tumor growth of the five successful xenografts obtained from three actinic keratoses (AKs) and two basal cell carcinomas (BCCs). Xenograft growth was assessed weekly by measuring the longer diameter of the tumor mass (mm) by caliper from week 1 to 12 post-implantation. Pt=patient

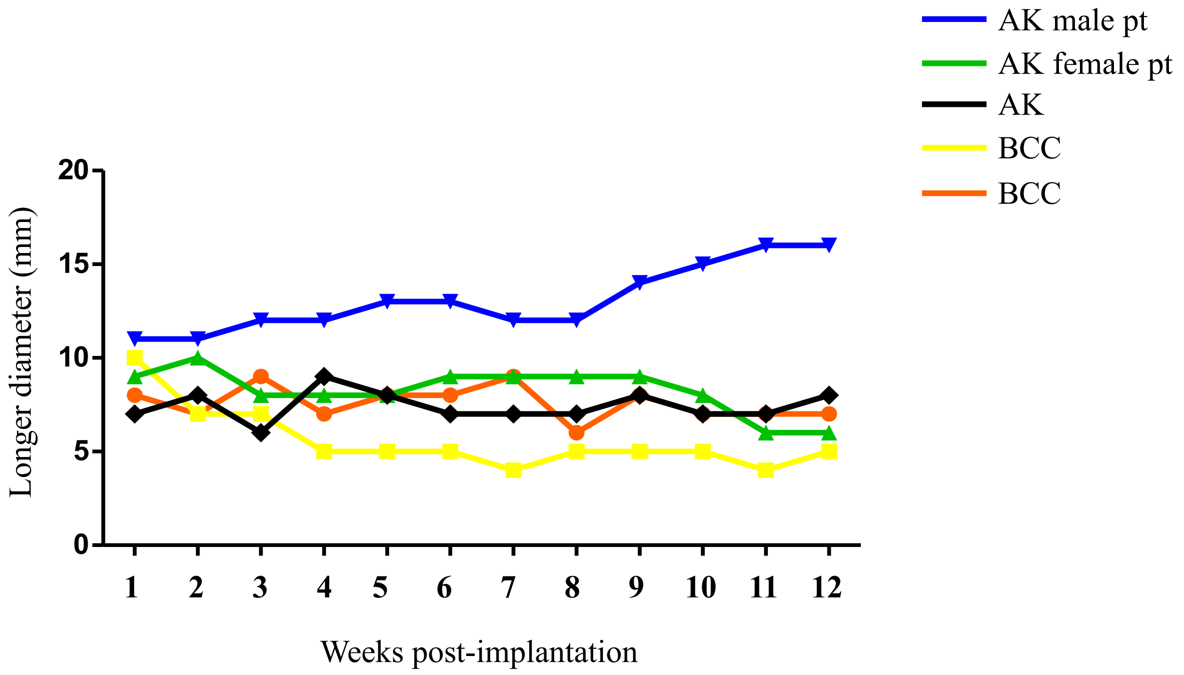

Supplement: Supplementary file 3 [file Image2.pdf]
